# The impact of the COVID-19 pandemic on harm reduction services in Spain

**DOI:** 10.1186/s12954-020-00432-w

**Published:** 2020-11-04

**Authors:** Camila A. Picchio, Jorge Valencia, Jason Doran, Tracy Swan, Marta Pastor, Elisa Martró, Joan Colom, Jeffrey V. Lazarus

**Affiliations:** 1 Barcelona Institute for Global Health (ISGlobal), Hospital Clínic, University of Barcelona, Barcelona, Spain; 2 Unidad Móvil de Reducción del Daño SMASD, Madrid, Spain; 3 Faculty of Public Health and Policy, London School of Hygiene and Tropical Medicine, London, UK; 4 Barcelona, Spain; 5 Comisión Ciudadana Antisida de Bizkaia, Bilbao, Spain; 6 Microbiology Department, Laboratori Clínic Metropolitana Nord, Hospital Universitari Germans Trias I Pujol, Institut D’Investigació en Ciències de La Salut Germans Trias I Pujol (IGTP), Badalona, Spain; 7 Centro de Investigación Biomédica en Red en Epidemiología Y Salud Pública (CIBERESP), Instituto de Salud Carlos III, Madrid, Spain; 8 Programme for Substance Abuse and for Prevention, Control and Treatment of HIV, STIs and Viral Hepatitis, Agency of Public Health of Catalonia, Barcelona, Spain

**Keywords:** COVID-19, Harm reduction, Healthcare utilisation, Health systems, Spain

## Abstract

**Background:**

Containment policies and other restrictions introduced by the Spanish government in response to the COVID-19 pandemic present challenges for marginalised populations, such as people who use drugs. Harm reduction centres are often linked to social services, mental health services, and infectious disease testing, in addition to tools and services that help to reduce the harms associated with injecting drugs. This study aimed to explore the impact of the pandemic on these services in four autonomous communities in Spain.

**Methods:**

This is a cross-sectional study that employed a seven-section structured survey administered electronically to 20 centres in July 2020. Data from the most heavily affected months (March–June) in 2020 were compared to data from the same period in 2019. Averages were calculated with their ranges, rates, and absolute numbers.

**Results:**

All 11 responding centres reported having had to adapt or modify their services during the Spanish state of alarm (14 March–21 June 2020). One centre reported complete closure for 2 months and four reported increases in their operating hours. The average number of service users across all centres decreased by 22% in comparison to the same period in the previous year and the average needle distribution decreased by 40% in comparison to 2019. Most centres reported a decrease in infectious disease testing rates (hepatitis B and C viruses, human immunodeficiency virus, and tuberculosis) for March, April, and May in 2020 compared to the previous year. Reported deaths as a result of overdose did not increase during the state of alarm, but 2/11 (18%) centres reported an increase in overdose deaths immediately after finalisation of the state of alarm.

**Conclusion:**

Overall, Spanish harm reduction centres were able to continue operating and offering services by adjusting operating hours. The number of overall service users and needles distributed fell during the Spanish state of alarm lockdown period, suggesting that fewer clients accessed harm reduction services during this time, putting them at greater risk of reusing or sharing injecting equipment, overdosing, acquiring infectious diseases with decreased access to testing or discontinuing ongoing treatment such as methadone maintenance therapy, hepatitis C treatment, or antiretroviral therapy.

## Background

On 31 December 2019, the first case of a novel coronavirus, SARS-CoV-2, was reported in Wuhan, China. By 30 January 2020, the World Health Organization (WHO) had declared a Public Health Emergency of International Concern, and on 11 March, declared a pandemic. COVID-19, the coronavirus disease caused by SARS-CoV-2, has spread to almost every country in the world and infected over 20 million people globally [1].

The effects of COVID-19, and the measures taken to contain it, have rippled across every aspect of society, magnifying existing inequalities among marginalised populations, such as people who use drugs (PWUD). PWUD face a range of legal, structural, and social barriers and high rates of morbidity and premature mortality [2]. Recognising the challenges faced by PWUD, in 2012 the United Nations proposed a comprehensive package of evidence-based harm reduction measures [3], which includes a number of services that aim to prevent the adverse effects of using drugs (Table 1).

**Table 1:** List of the United Nations-recommended services for people who use drugs

United Nations-recommended services for people who use drugs		Needle and syringe programmes
		Opioid substitution therapy
		HIV testing services
		Antiretroviral therapy for those living with HIV
		Prevention and treatment of sexually transmitted infections
		Condom programmes for people who inject drugs and their sexual partners
		Targeted information and education programmes
		Prevention, vaccination, diagnosis and treatment of viral hepatitis B and C
		Prevention, diagnosis and treatment of tuberculosis
		Community distribution of naloxone

*Sources:* WHO, UNODC, UNAIDS technical guide for countries to set targets for universal access to HIV prevention, treatment and care for injecting drug users, 2012 revision. Geneva: World Health Organization; 2013. Consolidated guidelines on HIV prevention, diagnosis, treatment and care for key populations, 2016 update. Geneva: World Health Organization; 2016

Harm reduction programmes often deliver or are linked with life-saving social programmes and medical and mental health services. Nonetheless, infection with the human immunodeficiency virus (HIV), hepatitis C virus (HCV) [4], and tuberculosis (TB) is prevalent and continues to spread among PWUD, partially driven by inadequate access to needle and syringe programmes (NSP) and opioid agonist therapy (OAT) [5], which, when combined, can lower the risk for HCV acquisition among PWUD by 74% [6] and also significantly lower HIV incidence [7, 8].

As a result of the COVID-19 containment measures, PWUD living with HIV, HCV or TB may face disruptions in access to antiretroviral therapy (ART), and direct-acting antivirals (DAAs) for HCV and TB treatment, which could drive drug resistance and/or lead to treatment failure. Similarly, in general, PWUD are at a greater risk of acquiring these infections and, as a result of the containment measures, may face disruptions in access to a timely diagnosis.

PWUD face an additional risk of infection with COVID-19 than people who do not use drugs [9], and could suffer from associated poor health outcomes. Drug use can involve close contact while sharing and/or reusing syringes and other paraphernalia and injecting in groups. Common co-morbidities (HCV [10], and chronic obstructive pulmonary disease (COPD) [11]) among PWUD are linked to adverse outcomes of COVID-19. In addition, COVID-19 mortality is associated with older age and smoking [12], both of which are common in PWUD [13, 14].

Across Europe, the containment policies that have been put in place to mitigate the spread of COVID-19 include strict physical distancing measures. Everyday services for PWUD had to change their opening hours or were shut down until further notice, including some health and social services [15, 16]. Some PWUD have been left without access to necessary services that allow them to protect themselves against, get tested, and seek treatment for bloodborne viruses (BBV) and TB; lower their risk of overdose; check the quality of the drugs they are using; and obtain necessary medical and mental healthcare, including for emergencies.

In Spain, use of alcohol and drugs was one of the top five contributors to disability-adjusted life years (DALYs) in 2016 [17]. Cocaine (39%), cannabis (29%), and heroin (24%) were the primary drugs resulting in treatment commencement in Spain and 58,749 clients received opioid substitution therapy (OST) in 2017 [18]. Of those who were admitted for addiction treatment in Spain in 2017, 14.3% began treatment for heroin use [19]. Spain has had a National Plan on Drugs [Plan Nacional Sobre Drogas (PNSD)] [20] since July 1985 and provides guidance on policies on drug use and drug dependence. The services offered to PWUD in Spain vary widely across the country’s 17 autonomous communities [21]. In 2017, 137 facilities for drug dependency, financed through the public and private sectors, were reported in Spain [19]. While harm reduction centres have been reported in 13 of the 17 autonomous communities, often these centres have insufficient data or resources for proper monitoring of outcomes, including the prevalence of infectious diseases among their clients.

In order to better understand the effect of the COVID-19 pandemic on harm reduction service provision in Spain, we report service utilisation, operating hours, and provision of harm reduction services such as OST, DAAs, and ART, infectious disease testing [hepatitis B and C viruses (HBV, HCV), human immunodeficiency virus (HIV), and tuberculosis (TB)], and mental health services during the pandemic and for the same period one year earlier.

## Methodology

### Study design

We conducted a cross-sectional study that employed a seven-section structured survey administered electronically to one focal point per centre. The survey was followed up to confirm results and clarify any uncertainties.

The survey consisted of questions regarding care provision, service user demographics, testing practices for infectious diseases (HBV, HCV, HIV and TB), reported prevalence of overdose, issues affecting PWUD during the Spanish state of alarm, and the relevant government responses to the pandemic for PWUD. Four key variables were collected from March to June 2019 and the same period in 2020 and included infectious disease testing and number of positive results, number of methadone treatments distributed, number of needles and syringes distributed, and the number of people commencing DAA treatment and ART. Data were also collected on the number of service users for January–June 2019 and the same period in 2020. One open-ended question was included to allow for elaboration on these topics.

All answers were dichotomous (yes/no) except for the questions on issues affecting PWUD during the Spanish state of alarm (Not a problem, somewhat a problem, neutral, problematic, very problematic) and the government response (excellent, above average, average, below average, bad), which were collected on a 5-point Likert scale. Prevalence of infectious diseases and the number of tests performed, number of total users, sex and nationality distribution, and the number of needles and syringes distributed were collected as continuous variables.

### Population and sample

Participants were harm reduction service providers who have been providing services in Spain since at least January 2019.

In 2017, 137 harm reduction centres were active in Spain. A harm reduction centre, as defined by the Spanish National Plan on Addictions (2017–2024) [20], includes all of the policies, strategies and programmes that ultimately aim is to lessen the negative effects of substance use (without necessarily aiming to achieve a reduction in their use) either at an individual level, or in families, both in the area of treatment and prevention, and social incorporation. Harm reduction centres in Spain may be comprehensive or focus on specific aspects that reduce the harm associated with drug consumption.

Twenty harm reduction centres were contacted in four Spanish autonomous communities with the highest prevalence of PWUD: the Basque Country, Catalonia, Madrid, and Valencia.

### Data analysis

Standard descriptive statistics were used to examine the distribution of key outcomes variables. Means and ranges were calculated for continuous variables and percentages and numbers for categorical variables. These data were used to describe the study respondents and address the descriptive research question. To calculate testing rates, the number of users in each centre was used as the denominator.

### Ethical considerations

No personal identifying information was collected and the IRB confirmed that no ethical approval was required. In an introductory email sent individually to each harm reduction centre focal point, information on the study and its objectives were provided. All data were reported at the centre level and data were kept on a password-protected Excel file.

## Results

Thirteen of the 20 (65%) centres contacted returned the completed survey: the Basque Country (*n* = 4), Catalonia (*n* = 6), Madrid (*n* = 2), and Valencia (*n* = 1). All 13 centres provide NSP and four (30%) have a supervised injecting room. All centres offer mental health services by connecting users to established programmes, including other non-governmental organisations (NGOs) (*n* = 4), referring them to established services in the public system (*n* = 7), or providing the services that are integrated into their centre (*n* = 2). A summary of the main services offered by the 11 centres is described in Table 2. All centres (*n* = 13) reported having had to adapt procedures in their centres by making mask-use mandatory, setting up socially distanced spaces, limiting capacity, and/or installing Plexiglas in order to guarantee the continuation of their activities during the Spanish COVID-19 state of alarm. Of these, two centres shut down during this period.

**Table 2:** Description of harm reduction services regularly provided by centres and number of users per centre (*n* = 11) during the first 6 months of the years 2019 and 2020, Spain (*n* = 11)

Centre (city name)	*n*. users 2019 (Jan–June)	*n*. users 2020 (Jan–June)	NSP	SIR	Naloxone distribution	Education services	Mental health services	OST	HCV DAA	HIV ART	HBV testing	HCV testing	HIV testing	TB testing
Centre 1 (Badalona)	265	274	✓		✓	✓	✓							
Centre 2 (Bilbao)	923	721	✓	✓		✓	✓					✓		✓
Centre 3 (Madrid)	4528	1945	✓		✓	✓	✓	✓	✓	✓	✓	✓	✓	✓
Centre 4 (Madrid)	892	1090	✓		✓	✓	✓	✓	✓	✓	✓	✓	✓	✓
Centre 5 (Vitoria-Gasteiz)	77	60	✓				✓	✓					✓	
Centre 6 (Bilbao)	1267	1080	✓			✓	✓		✓	✓	✓	✓	✓	
Centre 7 (Reus)	394	398	✓		✓	✓	✓	✓						
Centre 8 (Valencia)	764	687	✓			✓	✓	✓	✓	✓	✓	✓	✓	✓
Centre 9 (Bilbao)	1365	679	✓			✓	✓							
Centre 10 (El Prat de Llobregat)	2652	3351	✓		✓	✓	✓	✓		✓		✓	✓	
Centre 11 (Barcelona)	N/A	N/A	✓	✓	✓	✓	✓		✓		✓	✓	✓	✓
Centre 12 (Barcelona)	N/A	N/A	✓	✓	✓	✓	✓	✓	✓		✓	✓	✓	✓
Centre 13 (Sant Adrià de Besos)	6166	3912	✓	✓	✓	✓	✓		✓			✓	✓	
Total			13	4	8	12	13	7	7	5	6	9	9	6

*NSP* Needle and syringe programme, *SIR* supervised injecting room, *OST* opioid substation therapy, *DAA* direct acting antivirals, *HBV* hepatitis B virus, *HCV* hepatitis C virus, *HIV* human immunodeficiency virus (HIV), *TB* tuberculosis, *N/A* not available

The average age of clients in 2019 in the centres that provided data (*n* = 7) was 43.7 years (range: 40–47) and the average number of users in 12/13 centres in the same year was 1281 (range: 15–8748). All centres that responded (12/13) served more men (12,400 total, range: 13–6735) than women (2969 total, range: 2–2013). Of the 11 centres that provided data, Spanish nationals (9799 total, range: 11–5861) made up the majority of service users compared to those from other countries (5064 total, range: 4–2887).

### Impact of the COVID-19 pandemic on harm reduction operating hours and service users

Eleven out of thirteen (84.6%) centres reported that they were able to keep their centres open and operational during the Spanish state of alarm. One centre had to close in March and April 2020 but reopened on 27 May 2020 and the other closed from March until the end of the state of alarm (21 June 2020). The average weekly operating hours in 2019 for centres that reported these data (*n* = 9) was 52.4 h (range: 15–92) and in 2020 it was 47.4 h (range: 25–82). Four centres reduced their operating hours while the remaining five others increased their operating hours (Table 3).

**Table 3:** Operating hours and percentage change of centres with reported data (*n* = 9) pre-state of alarm and during

Centre (city name)	Weekly operating hours (pre-state of alarm)	Weekly operating hours (during state of alarm)	% Change
Centre 1 (Badalona)	15	25	+ 67%
Centre 2 (Bilbao)	52.5	66.5	+ 27%
Centre 3 (Madrid)	63	28	− 56%
Centre 4 (Madrid)	63	49	− 22%
Centre 5 (Vitoria-Gasteiz)	35	15	− 57%
Centre 6 (Bilbao)	66	84	+ 27%
Centre 7 (Reus)	N/A	N/A	N/A
Centre 8 (Valencia)	N/A	N/A	N/A
Centre 9 (Bilbao)	N/A	N/A	N/A
Centre 10 (El Prat de Llobregat)	29	42.5	+ 47%
Centre 11 (Barcelona)	56	35	− 37.5%
Centre 12 (Barcelona)	N/A	N/A	N/A
Centre 13 (Sant Adrià de Besos)	92	82	− 11%

**Fig. 1:**
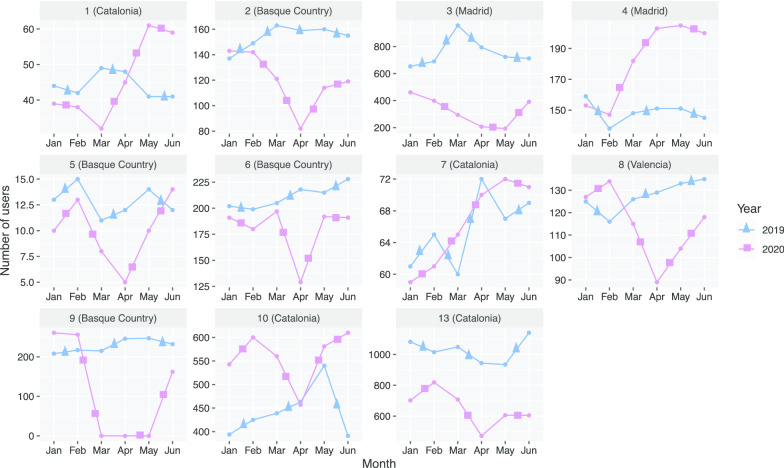
Time trend graphs describing number of users per centre that reported data (*n* = 11) during the first 6 months of 2019 and 2020. *Notes:* Free Y-scale axis utilised to visualise the relative differences

The average number of service users between the months of January–June 2019 in the centres (*n* = 11) was 292 (range: 11–1141) in comparison to the same months in 2020, which was 215 (range: 0–819). The difference in the overall average number of service users reflects a decrease of 26.4%. There was great variability among centres (Fig. 1).

### Impact of the COVID-19 pandemic on needle and syringe programmes and distribution of materials

**Fig. 2:**
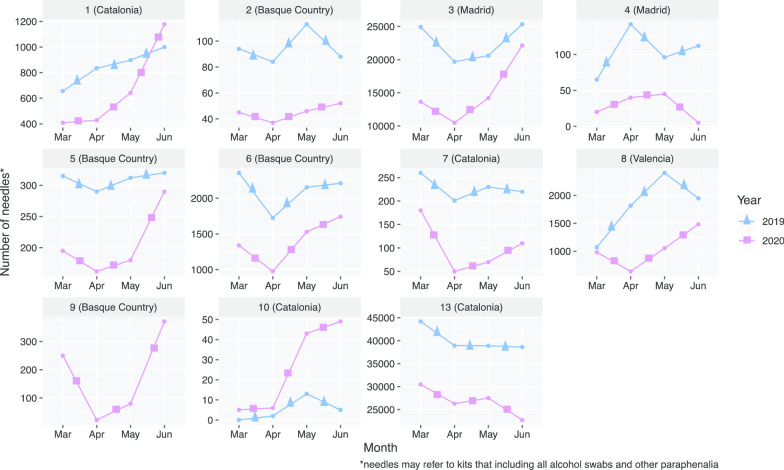
Time trend graphs describing the number of distributed needles in each centre that reported data (*n* = 11) between the months of March and June in 2019 and 2020. *Notes:* Free Y-scale axis utilised to visualise the relative differences

Ten of the 13 (76.9%) centres reported data on needle distribution for the months of March–June 2019 and 11/13 (84.6%) reported data for the same months in 2020. One centre did not have 2019 data available (Centre 9). March 2019 had the highest monthly average of needle distribution (7393) among the 10 centres that reported these data and April 2020 had the least number of needles distributed on average (3555). The total 4-month average of distributed needles was 6831. During the same months in 2020, the average number of needles distributed reduced by 40% (4140). There was variability between each centre and some reported an increase in needle distribution in the 2020 months compared to 2019 (Fig. 2).

### Impact of the COVID-19 pandemic on infectious disease testing

All centres reported testing for infectious diseases in situ. Six (46%) centres reported offering HBV testing and Mantoux tests for TB while nine (69%) offered HCV and HIV testing. Not all centres provided testing data despite reporting that they do provide them. Overall, most centres reported a decrease in testing rates for March, April, and May in 2020 compared to the year before for all infectious diseases. In some centres, HIV and HCV testing greatly increased in June 2020 in comparison to the year before and the month prior (May 2020). Testing rates for each centre for the months of March–June in 2019 and 2020 are described in Table 4.

**Table 4:** Infectious disease (HBV, HCV, HIV, and TB) testing rate per 1000 service users in March–June 2019 and March–June 2020

	Mar 19’	Mar 20’	Apr 19’	Apr 20’	May 19’	May 20’	Jun 19’	Jun 20’
	*HIV testing rates*							
Centre 3	50.2	23.8	35.3	29.0	52.6	72.5	49.2	97.2
Centre 4	40.5	38.5	211.9	0	112.6	4.9	27.6	0
Centre 6	58.5	30.5	36.7	0	41.9	46.9	65.8	193.7
Centre 8	63.5	52.2	85.3	0	90.2	19.2	14.8	67.8
Centre 10	2.8	1.8	0	0	0	0	0	0
Centre 13	7.6	14.1	9.5	0	39.6	13.2	35.9	23.1
	*HCV testing rates*							
Centre 2	0	0	0	0	0	0	0	8.4
Centre 3	50.2	23.8	35.3	29.0	52.6	72.5	49.2	97.2
Centre 4	40.5	38.5	211.9	0	112.6	4.9	27.6	0
Centre 6	58.5	30.5	36.7	0	41.9	46.9	65.8	193.7
Centre 8	63.5	52.2	85.3	0	90.2	19.2	14.8	67.8
Centre 10	2.8	1.8	0	0	0	0	0	0
Centre 13	7.6	14.1	9.5	0	39.6	13.2	35.9	23.1
	*HBV testing rates*							
Centre 3	29.3	20.4	21.4	0	26.3	0	28.1	71.6
Centre 4	20.3	11.0	19.9	0	0	0	0	0
Centre 6	0	5.1	0	0	0	5.2	0	20.9
Centre 8	39.7	26.1	54.3	0	22.6	0	7.4	0
Centre 10	2.8	1.8	0	0	0	0	0	0
*TB testing rates*								
Centre 2	0	0	0	0	0	8.8	0	16.8
Centre 3	8.4	13.6	2.5	0	5.5	0	7.0	25.6
Centre 4	40.5	27.5	72.8	0	38.7	0	20.7	10.0
Centre 8	7.9	8.7	38.8	11.2	22.6	0	22.2	0

Only centres that reported providing HBV, HCV, HIV, and TB testing are reflected in this table

### Impact of the COVID-19 pandemic on treatment administration (methadone, DAAs, ART)

Six (46%) out of 13 centres reported providing methadone on-site, and six (46%) centres provided DAAs and five (38%) provided ART, respectively. In the months of March–June 2019, 1163 clients in all centres received methadone versus 1422 in the same months in 2020, seeing a 22% increase in methadone distribution. In the six centres that reported distributing DAA therapy, 88 clients received DAAs in 2019 during the same months compared to 16 in 2020. One centre did not report this data for 2019. This represents an 82% decrease. In the five centres that reported ART distribution, 10 clients received ART in 2019 compared to 13 during the same months in 2020, showing a 30% increase.

### Impact of the COVID-19 pandemic on overdose and medical emergencies

Nine of the 13 (69%) centres responded that they did not see an increase in reported overdose deaths during the Spanish state of alarm, while one centre (7.6%) reported they did see an increase, another (7.6%) did not know, and two centres did not respond.

Two of the 13 (15.4%) centres responded that they saw an increase in reported overdose deaths immediately after the Spanish state of alarm ended. One centre (7.6%) did not know if overdose deaths increased, the remaining (61.5%) did not report an increase in overdose deaths in their centres, and two (15.4%) did not respond.

There were a total of 99 reported medical emergencies across all centres that responded (9/13) between March and June 2019 with a range of zero medical emergencies to 26 per month. During the months of March to June 2020, there were a total of 96 reported medical emergencies among the same centres, ranging from zero medical emergencies to 25 per month.

### Challenges faced by PWUD during the COVID-19 pandemic state of alarm in Spain

**Fig. 3:**
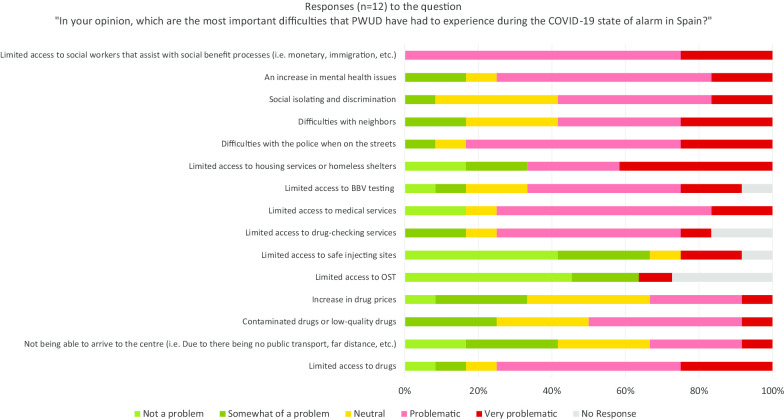
Distribution of responses describing the most important difficulties that PWUD had to experience during the COVID-19 state of alarm in Spain, 2020

Twelve (92%) centres responded to the questions regarding challenges faced by PWUD during the COVID-19 pandemic state of alarm. Those who responded to the survey reported that limited access to social workers who assist with social benefit processes was the most problematic issue affecting PWUD, with all 12 (100%) people surveyed responding that it was “problematic” or “very problematic”. Ten (83%) respondents also believed that difficulties with the police while on the street would be a “problematic” or “very problematic” issue for PWUD, in addition to an increase in mental health issues (8/12) and limited access to drug-checking services (6/12) (Fig. 3).

### Government response to the COVID-19 pandemic

The types of regional agencies responsible for managing and coordinating the COVID-19 response in their autonomous communities for PWUD vary. Of those that responded to the question (*n* = 12), the majority of centres said that the responsible entity performed average (*n* = 4) or below average (*n* = 4). One centre responded that their responsible administration did an excellent job and three (25%) reported that their agencies did better than average.

## Discussion

Pandemics expose weaknesses in societies and bring to the fore a country’s ability to respond and protect its most vulnerable people [22]. Measures to mitigate the effects of COVID-19 have had serious social and economic impacts; although they have affected everyone, the impact is largest among marginalised and vulnerable populations [23, 24]. This study set out to understand if, and to what extent, services for PWUD, an already vulnerable population, were affected during Spain’s COVID-19 state of alarm—and the potential consequences of pandemic-related disruptions.

Service closures and disruptions have been reported in the United States, western and eastern Europe, sub-Saharan Africa, and Latin America and the Caribbean [22, 25–28]. Our study found that all of the 13 participating harm reduction centres reported having had to modify or adapt their services. Overall, the average weekly operating hours across all centres decreased during Spain’s state of alarm; however, four of the centres (13 responded) actually increased their operating hours during this period. Although Spain was one of the most heavily affected countries in the European Union, its network of harm reduction centres showed resilience by adapting policy changes and innovative strategies to deliver essential services to PWUD. Similarly, the only community checkpoint offering services to men who have sex with men in Barcelona reported a closure of services to the public from 13 April to 18 May 2020 (personal communication). However, in an attempt to reduce the risks associated with a cessation of essential services to this population, only HIV and STI testing were interrupted during this time while administration of ART and PrEP continued with follow-up via telehealth.

Although these centres were largely able to stay open, they served a smaller number of people, and distributed fewer needles in 2020 than in 2019. This decline in NSP coverage could lead to an increased risk for needle reuse or sharing, and a corresponding increase in incidence of BBVs, though further research should investigate whether the decline corresponded to the reduction in the drug supply. In addition, as a result of reduced operating hours or closures, testing for infectious diseases and linkages to health and social service programmes were affected, with several centres operating at reduced hours or deprioritising testing, given that the health system was overwhelmed with COVID-19 cases. Although it may be too early to determine the impact of these reductions on the incidence of viral hepatitis, HIV or TB, this should be a priority for further research.

Harm reduction programmes reach people at high risk for infectious diseases. People who inject drugs are 22 times more likely to be living with HIV than the general population [29], representing nearly 20% of the estimated 15.6 million people who inject drugs globally. HCV is highly prevalent, with approximately 50% of PWUD currently or previously infected [4]. Timely diagnoses of these diseases in centres that are regularly used by, and accessible to, PWUD is imperative for individual and public health. A breach in these people-centred models of care [30] for PWUD could derail progress towards global goals and targets to eliminate viral hepatitis [31] and AIDS as threats to public health.

Additionally, PWUD face risks to their survival, health, and well-being, since many are unstably housed (including street homelessness). This social isolation increases the likelihood of overdose, which may be further compounded by lack of regular access to OAT and/or opioids due to COVID-19 prevention measures [26] that restrict movement in some settings. While our study did not report an overall increase in reported overdose deaths, we recognize that this can be a significant issue in other settings [32].

Despite this study reporting data from the state of alarm, which the Spanish government declared due to the ongoing COVID-19 pandemic, none of the surveyed centres offered COVID-19 testing during that time. Testing for the SARS-CoV-2 virus could be expanded into harm reduction services to include it through proper referral or in parallel through community-based screening. As point-of-care diagnostic methods continue to develop, testing in these settings should be considered. Harm reduction centres that have GeneXpert platforms (Cepheid Inc.) for HCV and TB testing available in their facilities or in community-based centres, should consider using this or other similar platforms for COVID-19 testing among their clients [33]. Early detection of COVID-19 could help mitigate possible complications caused by the virus among PWUD, who may have underlying chronic medical conditions that increase their risk of developing severe COVID-19 illness, such as COPD, asthma, cardiovascular diseases, and viral hepatitis [9]. PWUD are at risk for sepsis and endocarditis, and commonly have skin and soft tissue infections [34, 35] which, when left untreated, can lead to hospitalisation and even amputation, putting them at greater risk if infected with COVID-19.

Drug treatment services and low-threshold harm reduction services for PWUD are essential health services, which need to maintain operational under restricted conditions brought on by global crises, like pandemics [15]. Our study showed that this was possible in the Spanish context, where both government and non-governmental-run services adapted their opening hours. However, such health system resilience should not be left to chance or to the goodwill of service providers. Spain and other countries across Europe need to be prepared for the continuation of policies that may disrupt services during the current pandemic and/or future pandemics. In a recent study measuring the public’s perception of government COVID-19 pandemic response efforts, Spain was ranked low with 44 out of 100 points, indicating that the Spanish government needs to improve its communication with all of its constituents, and according to the results of the study, in particular, in protecting the welfare of vulnerable populations, which includes PWUD [36]. Pandemic preparations and response should include making the health system more people-centred through improved engagement and communication with all key stakeholders [37, 38], not least the clients of harm reduction services.

## Conclusion

Overall, consulted Spanish harm reduction centres were able to continue running and offering services by adjusting operating hours; only two centres completely shut down for a specific period of time. The number of overall service users and needles distributed fell during the Spanish lockdown period, suggesting that fewer clients accessed harm reduction services during that time. This may have resulted in greater risk of reusing or sharing injecting equipment, overdosing or acquiring infectious diseases, in addition to decreased access to testing or discontinuing ongoing treatment such as methadone maintenance therapy, hepatitis C treatment, or antiretroviral therapy. For centres that were able to remain open, and in some instances expand operating hours, lessons learned should be shared with the centres that were unable to continue offering services so that harm reduction services remain operational during the COVID-19 pandemic in the event of future pandemics.

## Data Availability

The datasets used and/or analysed during the current study are available from the corresponding author upon reasonable request.
